# Advances in MRI Techniques and Applications

**DOI:** 10.1155/2015/139043

**Published:** 2015-09-02

**Authors:** Zhengchao Dong, Trevor Andrews, Chuanmiao Xie, Takeshi Yokoo

**Affiliations:** ^1^Division of Translational Imaging and MRI Unit, College of Physicians and Surgeons, Columbia University and New York State Psychiatric Institute, New York, NY, USA; ^2^MRI Center for Biomedical Imaging, Department of Radiology, University of Vermont, Burlington, VT, USA; ^3^Department of Medical Imaging and Interventional Radiology, Cancer Center, Sun Yat-Sen University, Guangzhou, Guangdong, China; ^4^Advanced Imaging Research Center, University of Texas Southwestern Medical Center, Dallas, TX, USA

Magnetic resonance imaging (MRI) has been playing an increasingly important role in biomedical research and in clinical diagnosis. The techniques of MRI have experienced rapid development and found wide applications in recent years. The technical development is marked not only by the improvement and optimization of conventional MR imaging techniques but also by the emergence of new pulse sequences such as CEST-MRI (Chemical Exchange Saturation Transfer MRI) and DWIBS (Diffusion-weighted Whole-body Imaging with Background body signal Suppression) and by new techniques such as compressed sensing MRI and MR fingerprinting. The wide proliferation of MRI techniques has led to ever-increasing applications of MR imaging and enormous new findings in basic biomedical research as well as clinical sciences. This special issue aims at reflecting the advances in MR imaging techniques and applications.

After rigorous review procedures, the selected papers in this special issue demonstrate how broad the field of MRI has become since Paul Lauterbur's classic paper in 1973. The types of papers include review articles as well as original research. Within this issue are papers that study not only data acquisition and reconstruction methods but also specialized analysis methods and new combinations of MR with therapeutic and interventional techniques. The imaging methods cover structural MRI, functional MRI, diffusion-weighted imaging, and diffusion tensor imaging. The applications addressed are similarly wide reaching, containing cancer, liver diseases, brain function, and so forth.

Of particular note, this special issue contains several papers on perhaps the most common use of MRI in MR research centers: functional MRI (fMRI) of the brain. Neuronal activation in the brain results in local increase in the oxygen-enriched blood in capillaries associated with the signal change measured in traditional BOLD-based fMRI. One paper (J. Chung et al.) examines the fusiform face region and the fundamental confoundedness of the signal of blood from large vessels causing a mismatch between the localization of neuronal activation and that of fMRI signal. Once considered to be simply “physiological noise,” certain characteristic fluctuations in the fMRI signal during rest have been shown to be associated with specific neuronal networks. Due to the practical appeal of fMRI without tasks, we are including two review papers reflecting the recent great interest in clinical methods for “resting state” fMRI, one for multiple sclerosis (E. Sbardella et al.) and the other for psychiatric disorders (X. Zhan et al.). In a related paper (X. Li et al.), a temporal decomposition method is presented which decomposes a single brain functional network into several modes to explore dynamic brain functional networks in a continuous, “state-related,” “finger-force feedback” fMRI experiment. Finally, two papers are included which highlight some of the many ways in which advanced data acquisition (D. Kang et al.) and image reconstruction methods (P. K. Han et al.), such as segmented echo-planar imaging (EPI) and compressed sensing, can help to overcome some of the image quality obstacles of fMRI at high field strength.

Two other neuro-MRI studies are presented as well regarding areas of strong interest in MR: diffusion tensor imaging (DTI) and MR guidance. In the first paper (T.-K. Truong et al.), a new method is presented for the correction of eddy current-induced echo-shifting effect that produces three types of artifacts, namely, the eddy current induced signal loss, the artificial signal modulation due to eddy current-induced erroneous *T*_2_^*∗*^ weighting, and artificial signal elevation associated with partial Fourier reconstruction. The second paper (E. Vaghefi et al.) presents an MRI-based technique to guide the noninvasive transcranial brain stimulation without the use of a neuronavigation system.

As MR has matured technically primarily for neuroapplications, it has also proven to be increasingly useful for body imaging. An example is the technique of real-time MR thermometry guidance for ultrasound ablation of uterine fibroids, in which excessive skin heating has been an obstacle. To address this issue, the feasibility and safety of utilizing a water-cooled device in contact with the skin are proposed and examined in a paper (M. Ikink et al.). Another “hot topic” in body MR has been the study of liver fibrosis, using a variety of MR methods (e.g., MR elastography and DWI). In this issue, a paper (T. Yokoo et al.) is included which examines the use of a combined Gd and USPIO contrast agent together with texture analysis to better depict the reticular signal abnormalities associated with fibrosis. In a review of MRI for Crohn's Disease (K. Yoon et al.), a variety of methods are presented for inflammatory bowel disease including endoluminal and intravenous contrast agents, DWI, dynamic bowel motility imaging, and MR spectroscopy of fecal and urine samples. Finally, MR-based molecular imaging is presented in a review paper (J. H. Kim et al.) focusing on labelling stem cell with nanoparticles in urology to evaluate migration and survival of transplanted stems cells in prostate cancer and bladder dysfunction models.

With the many technical advances in the field, several MR methods have demonstrated their utility in terms of oncology. One paper (I. Thomassin-Naggara et al.) reviews the impact of perfusion and diffusion MRI and a new diagnostic MR scoring system (Adnex MR) upon the preoperative diagnostic accuracy in women with possible ovarian cancer. The utility of combining structural, perfusion, diffusion, and MR spectroscopy data is also presented in this issue for tumour relapse prediction using multiparametric analysis in glioblastoma patients (E. Vaghefi et al.). The use of MR (using diffusion MR and/or dynamic contrast enhancement) to predict the response to neoadjuvant therapy is presented in two papers (G.-Y. Zhang et al. and M. Petrillo et al.) focusing on nasopharyngeal carcinoma and rectal cancer, respectively.

In summary, the papers collected in this special issue cover a wide range of topics that are on the frontier of the MRI techniques and their applications.

